# Treatment Disparities in Georgia: Methamphetamine Use Disorder

**DOI:** 10.7759/cureus.54885

**Published:** 2024-02-25

**Authors:** David O Ossai, Bolaji Yoade, Oluwatoyin Busari, Princess Agwu, Tinuoye Adewoye, Fanta Kalle, Rosemary Agwu, Ayodele Atolagbe, Stanley Nkemjika

**Affiliations:** 1 Department of Public Health, Georgia State University, Atlanta, USA; 2 Psychiatry and Behavioral Sciences, Interfaith Medical Center, Brooklyn, USA; 3 Psychiatry, University Hospitals Cleveland Medical Center, Case Western Reserve University, Cleveland, USA; 4 Nursing Science, Abia State University, Uturu, NGA; 5 Medicine, Waubonsie Valley High School, Aurora, USA; 6 Psychiatry and Behavioral Sciences, Edward Via College of Osteopathic Medicine, Auburn, USA; 7 Medical Radiography and Radiological Sciences, University of Nigeria, Nsukka, NGA; 8 Psychiatry and Behavioral Sciences, Kingsbrook Jewish Medical Center, Brooklyn, USA

**Keywords:** methamphetamine, georgia, disparities, treatment, sociodemographic

## Abstract

Background: Methamphetamine use disorder (MUD) is becoming more of a public health issue in Georgia State, with health and social effects affecting both people and communities. This study aimed to investigate attributes that may affect the accessibility of treatment among the methamphetamine-use population in the state of Georgia.

Methods: We utilized the Treatment Episode Data Set - Discharges (TEDS-D) for 2016-2020 in Georgia, comprising participants with MUD (175,270). We utilized descriptive statistics and inferential techniques to ascertain the relationship between variables. Multiple logistic regression was used to control for confounding variables at a 95% confidence interval.

Results: This study's findings showed individuals aged 25-49 years had 1.8 times higher odds of getting treatment for methamphetamine use compared to those aged 12-24 years (adjusted odds ratio (AOR) = 1.8; 95% CI: 1.50-2.16). Alaska Native individuals (Aleut, Eskimo, and Indian) had 7.07 times higher odds of receiving treatment than Asian or Pacific Islander individuals (AOR = 7.07; 95% CI: 2.02-24.67). Compared to Asian or Pacific Islander individuals, Black or African American individuals had 12.11 times higher odds of receiving treatment (AOR = 12.11; 95% CI: 9.37-15.66), while White individuals had 6.82 times higher odds of getting treatment (AOR = 1.09; 95% CI: 0.86-1.37).

Conclusion: MUD treatment disparity challenges are revealed in our study, emphasizing the critical need for focused intervention programs.

## Introduction

Methamphetamine (meth) is a highly addictive stimulant that impacts the central nervous system by increasing the quantity of dopamine within the brain [1]. Meth is developed from amphetamine, considered the parent drug since the 20th century [2,3]. Since its development, the United States (US) Drug Enforcement Administration categorized it as a Schedule II stimulant; thus, meth can only be considered available via non-refillable prescriptions, which are mainly prescribed for the medical management of attention deficit hyperactivity disorder (ADHD) [4]. The number of people using meth is rapidly growing, indicating how spread the practice is becoming over the years. As of 2017, about 1.6 million people in the USA were established to use meth following a study by the National Survey on Drugs and Health [5]. In addition, the use of meth has not been without death cases as evidence in the literature suggests that death from its overdose is the second highest mortality after cocaine, among the psychostimulant family [6]. Georgia has not been spared either in these statistics.

According to 2017 statistics, Georgia registered an increase in the use of meth, which can be attributed to various factors like interrelated burdens of stress, mental illness, housing instability, geographic mobility, and poverty [7]. Additionally, meth use is more prevalent in areas with more significant socioeconomic hardship and less access to resources. Furthermore, the role of the US drug policy and criminal justice systems have been fundamental in the rising use of meth in Georgia State due to increased demand in the black market, with equally increasing cases of recidivism among individuals using [8,9]. Other notable reasons stem from its spread, which has been propelled by the comparatively low cost of production and significant revenue for traffickers, resulting in a surge in availability and use [10]. According to Kidd et al. (2019), younger people are more likely to use meth, especially those who are in their late teens and early 20s. In recent years, there has been a change, with more middle-aged and older persons seeking addiction treatment for meth [11]. This demographic shift indicates a widening user base and the need for tailored prevention and intervention efforts for different age groups.

Similarly, meth use prevalence in the US varies across several ethnic and racial groupings as studies have indicated that specific minority populations, such as Native Americans and Pacific Islanders, use drugs more frequently [12,13]. These variances may be attributed to socioeconomic variables, historical trauma, and social inequality, emphasizing the necessity of addressing underlying causes to battle the surge in meth usage effectively. Hoots et al. (2020) noted that meth use disorder is a complicated issue that is impacted by numerous individual, societal, and environmental factors [14]. Developing focused prevention and intervention methods requires an awareness of the risk variables related to meth use. Thus, identifying vulnerable populations can also aid in resource allocation and health inequalities reduction. However, there remains a dearth of literature on the treatment attributes of the meth use population. Similarly, although meth is one of the popular substances in Georgia, there is no information on the utilization and accessibility of treatment resources for this population. Hence, we aim to understand the sociodemographic characteristics of the population seeking treatment for meth use disorder in the state of Georgia.

## Materials and methods

Data and analytic sample

Data for 2010-2020 were obtained from the Substance Abuse and Mental Health Services Administration's (SAMHSA) Treatment Episode Data Set - Discharges (TEDS-D), a national dataset of annual admissions and discharges for substance use disorder (SUD) treatment facilities administered by SAMHSA. All 50 states (including Washington D.C. and Puerto Rico) in the US collect and submit Treatment Episode Data Set (TEDS) data to the Federal Government. TEDS is estimated to include 83% of eligible drug and alcohol treatment admissions across the USA. TEDS-D reporting facilities receive state alcohol and drug agency funds, including Federal Block Grant funds, to provide SUD treatment. Each observation in the data is a "substance use treatment episode" and discharge record. The dataset includes records on admissions of individuals aged 12 years or older; admission demographics (age, sex, race/ethnicity, and employment status); and substance abuse characteristics (such as substances used, age at first use, frequency of use, and number of prior admissions). This study focused on the total population of methamphetamine use disorder (MUD) patients enrolled in TEDS-D for 2016-2020 in Georgia (175,270). The dataset was comprehensive and included patients who were admitted to SUD treatment facilities in Georgia. Additionally, the dataset was used to identify factors related to this first treatment admission as far as recent analysis. This study was limited to respondents who provided information on their meth use on admission.

Measures

Independent Variables

Independent variables included individual demographics like client age, biological sex, marital status, race, years of education, employment status, and homeless status.

Dependent Variable

Respondents were based on the documented diagnosis of meth use disorder as the response was "Yes" or "No."

Data analysis

We summarized the data on a continuous scale with mean, standard deviation, range, and median. The categorical data were summarized with count and percentage. We used chi-square tests to see if there were any relationships and differences between the groups as we got statistically significant estimates. We also used univariate and multivariate logistic analysis to see if there was an unadjusted or adjusted odds relationship. We assumed there was some confounder if the odds ratio went up by more than 10%, and they were added to the logistic regression model in the end (age was not statistically significant and was taken out). We also adjusted for any potential confounders by including some covariates based on what was known from the literature. We did not consider any weighted variables or missing values in the survey, so we kept the statistical rigor and did not delete any unwanted observations in the person-level files. All the analyses and graphics were done using SAS software 9.4, developed by the SAS Institute Inc. (Cary, NC), to handle complex design models. P-values were < 0.05 for all the analyses in this study.

## Results

Table 1 shows the descriptives of the study population (n = 175,270). Most individuals (63.55%) fell within the age range of 25-49 years. Of the individuals, 10.98% were aged between 12 and 24 years. Of the individuals, 25.47% were aged 50 years and above. Furthermore, most of the study population comprised individuals who identified as Black or African American (59.23%) and Asian or Pacific Islander (38.65%). A small percentage of the population identified as White (1.55%), Alaska Native (0.28%), or American Indian (0.29%). The study population was roughly evenly distributed by gender; 59.77% were male and 40.23% were female. Most individuals were unemployed (63.42%) or not in the labor force (22.29%). A smaller proportion of the population worked either full-time (8.68%) or part-time (5.61%). The most significant proportion of individuals fell under the "Never Married" (64.24%) group. Of the individuals, 11.81% were currently married ("Now Married") and 23.95% of the individuals were classified as "Separated."

**Table 1:** Characteristics of the study population (n = 175270) GED: General Educational Diploma; BA: Bachelor of Arts; BS: Bachelor of Science; HMO: Health Maintenance Organization.

Variable	n	Percent
Age		
12-24	19250	10.98
25-49	111376	63.55
>50	44644	25.47
Race		
Alaska Native	476	0.28
American Indian	494	0.29
Asian or Pacific Islander	66122	38.65
Black or African American	101345	59.23
White	2657	1.55
Gender		
Male	104696	59.77
Female	70468	40.23
Employment		
Full-time	12585	8.68
Part-time	8128	5.61
Unemployed	91928	63.42
Not in the labor force	32314	22.29
Marital status		
Never married	108913	64.24
Now married	20027	11.81
Separated	40608	23.95
Education		
Less than one school grade, no schooling, nursery school, or kindergarten to grade 8	13102	8.21
Grades 9-11	43041	26.97
Grade 12 or GED	65845	41.26
1-3 years of college, university, or vocational school	31035	19.45
Four years of college, university, BA/BS, some postgraduate study, or more	6580	4.12
Source of income/support		
Wages/salary	10676	54.99
Public assistance	513	2.64
Retirement/pension, disability	7015	36.13
Other	1210	6.23
Living arrangements		
Homeless	21086	13.39
Dependent living	25139	15.97
Independent living	111203	70.64
Type of treatment/setting		
Detox, 24-hour hospital inpatient	19640	21.39
Detox, 24-hour, free-standing residential	4699	5.12
Rehab/residential, hospital (non-detox)	67490	73.5
Number of arrests 30 days before admission		
None	166883	95.39
Once	7428	4.25
Two or more times	633	0.36
Health insurance		
Private insurance, Blue Cross/Blue Shield, HMO	2408	3.35
Medicaid	7302	10.17
Medicare, other (TRICARE, CHAMPUS)	4385	6.11
None	57719	80.37

Furthermore, the highest number of individuals (41.26%) had completed grade 12 or obtained a General Educational Diploma (GED). Of the individuals, 26.97% had education levels ranging from grades 9 to 11. A smaller proportion of the population had either no schooling, kindergarten, or nursery school to grade 8 (8.21%), one to two years of college, university, or vocational school (19.45%), or four years of college, postgraduate study, or more (4.12%). In addition, the most significant proportion of the study population (70.64%) was classified as living independently, while 15.97% of the individuals were in a dependent living situation, and 13.39% of the individuals were classified as homeless. Moreover, the majority of individuals (73.5%) received treatment in a "Rehab/residential, hospital (non-detox)" setting. Of the individuals, 21.39% were admitted to a "Detox, 24-hour, hospital inpatient" setting. A smaller proportion of the population (5.12%) received treatment in a "Detox, 24-hour, free-standing residential" setting (see Table 1 for other results). Table 2 demonstrates the similar characteristics of the study population stratified by meth use status.

**Table 2:** Study population stratified by methamphetamine use status GED: General Educational Diploma; BA: Bachelor of Arts; BS: Bachelor of Science; HMO: Health Maintenance Organization.

Variable	No meth use		Meth use		
	Frequency	Percent	Frequency	Percent	Prevalence (%)
Age					
12-24	17369	90.23	1881	9.77	1.07
25-49	97262	87.33	14114	12.67	8.05
>50	43102	96.55	1542	3.45	0.88
Race					
Alaska Native	451	94.75	25	5.25	0.01
American Indian	453	91.7	41	8.3	0.02
Asian or Pacific Islander	65041	98.37	1081	1.63	0.63
Black or African American	85489	84.35	15856	15.65	9.27
White	2371	89.24	286	10.76	0.17
Gender					
Male	96331	92.01	8365	7.99	4.78
Female	61307	87	9161	13	5.23
Employment					
Full-time	11106	88.25	1479	11.75	1.02
Part-time	7251	89.21	877	10.79	0.61
Unemployed	80844	87.94	11084	12.06	7.65
Not in the labor force	31300	96.86	1014	3.14	0.7
Marital status					
Never married	99080	90.97	9833	9.03	5.8
Now married	17715	88.46	2312	11.54	1.36
Separated	35900	88.41	4708	11.59	2.78
Education					
Less than one school grade, no schooling, nursery school, or kindergarten to grade 8	11640	88.84	1462	11.16	0.92
Grades 9-11	38348	89.1	4693	10.9	2.94
Grade 12 or GED	58731	89.2	7114	10.8	4.46
1-3 years of college, university, or vocational school	28691	92.45	2344	7.55	1.47
Four years of college, university, BA/BS, some postgraduate study, or more	6164	93.68	416	6.32	0.26
Source of income/support					
Wages/salary	8901	83.37	1775	16.63	9.14
Public assistance	401	78.17	112	21.83	0.58
Retirement/pension, disability	6488	92.49	527	7.51	2.71
Other	991	81.9	219	18.1	1.13
Living arrangements					
Homeless	19285	91.46	1801	8.54	1.14
Dependent living	21030	83.65	4109	16.35	2.61
Independent living	101041	90.86	10162	9.14	6.46
Type of treatment/setting					
Detox, 24-hour hospital inpatient	17979	91.54	1661	8.46	1.81
Detox, 24-hour, free-standing residential	3579	76.17	1120	23.83	1.22
Rehab/residential, hospital(non-detox)	57185	84.73	10305	15.27	11.22
Number of arrests 30 days prior to admission					
None	150021	89.9	16862	10.1	9.64
Once	6817	91.77	611	8.23	0.35
Two or more times	585	92.42	48	7.58	0.03
Health insurance					
Private insurance, Blue Cross/Blue Shield, HMO	2028	84.22	380	15.78	0.53
Medicaid	6563	89.88	739	10.12	1.03
Medicare, other (e.g. TRICARE, CHAMPUS)	3844	87.66	541	12.34	0.75
None	48249	83.59	9470	16.41	13.19

Table 3 presents the results of a logistic regression analysis predicting treatment for meth use based on various demographic factors. In the context of age, individuals aged 25-49 years had 1.8 times higher odds of getting treatment for meth use compared to those aged 12-24 years (adjusted odds ratio (AOR) = 1.8; 95% CI: 1.50-2.16). Individuals over 50 (greater than 50 years) had 0.86 times lower odds of getting treatment than those aged 12-24 years (AOR = 0.86; 95% CI: 0.65-1.14). Regarding education status, individuals with grades 9-11 had 0.88 times lower odds of getting treatment compared to those with no schooling, kindergarten, or nursery school to grade 8 (AOR = 0.88; 95% CI: 0.67-1.16). Individuals with grade 12 (or GED) education had 0.69 times lower odds of getting treatment compared to those with less than no schooling, kindergarten, or nursery school to grade 8 (AOR = 0.69; 95% CI: 0.54-0.90). Furthermore, individuals with one to three years of college, university, or vocational school education had 0.39 times lower odds of receiving treatment than those with less than one school grade, no schooling, nursery school, or kindergarten to grade 8 (AOR = 0.39; 95% CI: 0.30-0.52). In addition, individuals with four years of college, postgraduate study, or more education had 0.24 times lower odds of getting treatment compared to those with no schooling, kindergarten, or nursery school to grade 8 (AOR = 0.24; 95% CI: 0.16-0.37).

**Table 3:** Logistic regression predicting treatment in methamphetamine use based on age, education, marital status, employment status, race, primary source of income, and the type of treatment or setting at admission Reference key: ^a^ = 12-24 years; ^b^ = less than one school grade, no schooling, nursery school, or kindergarten to grade 8; ^c^ = not married; ^d^ = never employed; ^e^ = Asian or Pacific Islander; ^f^ = wages/salary; ^g^ = rehab/residential, hospital (non-detox). GED: General Educational Diploma; BA: Bachelor of Arts; BS: Bachelor of Science.

Variables	Point estimate	Confidence interval
Age		
25-49 years ^a^	1.8	1.50-2.16
>50 years ^a^	0.86	0.65-1.14
Education status		
Grades 9-11 ^b^	0.88	0.67-1.16
Grades 12 (or GED) ^b^	0.69	0.54-0.90
1-3 years of college, university, or vocational school ^b^	0.39	0.30-0.52
Four years of college, university, BA/BS, some postgraduate study or more ^b^	0.24	0.16-0.37
Marital status		
Now married^ c^	0.87	0.73-1.03
Separated^ c^	1.12	0.97-1.29
Employment status		
Full-time^ d^	0.98	0.84-1.14
Part-time ^d^	0.94	0.79-1.12
Not in the labor force ^d^	0.09	0.01-0.69
Race		
Alaska Native (Aleut, Eskimo, Indian)^ e^	7.07	2.02-24.67
American Indian (other than Alaska Native) ^e^	3.87	1.34-11.15
Black or African American^ e^	12.11	9.37-15.66
White^ e^	6.82	4.55-10.22
The primary source of income/support		
Public assistance^ f^	1.57	1.16-2.14
Retirement/pension, disability ^f^	0.87	0.67-1.13
Other ^f^	1.09	0.86-1.37
Type of treatment or service setting at admission		
Detoxification, 24-hour service, hospital inpatient^ g^	<0.001	<0.001->999.99
Detoxification, 24-hour service, free-standing residential ^g^	1.18	0.85-1.63

Concerning marital status, married individuals had 0.87 times lower odds of getting treatment than those who had never been married (AOR = 0.87; 95% CI: 0.73-1.03). Separated individuals had 1.12 times higher odds of getting treatment than those who had never been married (AOR = 1.12; 95% CI: 0.97-1.29). Regarding employment status, full-time employees had similar odds of getting treatment compared to unemployed individuals (AOR = 0.98; 95% CI: 0.84-1.14). Part-time employees had similar odds of receiving treatment compared to unemployed individuals (AOR = 0.94; 95% CI: 0.79-1.12), while individuals not in the labor force had 0.09 times lower odds of getting treatment than unemployed individuals (AOR = 0.09; 95% CI: 0.01-0.69). In the context of race, Alaska Native individuals (Aleut, Eskimo, and Indian) had 7.07 times higher odds of receiving treatment than Asian or Pacific Islander individuals (AOR = 7.07; 95% CI: 2.02-24.67). Compared to Asian or Pacific Islander individuals, American Indian individuals (other than Alaska Natives) had 3.87 times higher odds of getting treatment (AOR = 3.87; 95% CI: 1.34-11.15), Black or African American individuals had 12.11 times higher odds of receiving treatment (AOR = 12.11; 95% CI: 9.37-15.66), while White individuals had 6.82 times higher odds of getting treatment (AOR = 1.09; 95% CI: 0.86-1.37).

In the context of the primary source of income, individuals receiving public assistance had 1.57 times higher odds of accessing treatment than those on salary/wages, which was statistically significant (AOR = 1.57; 95% CI: 1.16-2.14). Individuals receiving retirement/pension/disability support (AOR = 0.87; 95% CI: 0.67-1.13) or other types of income (AOR = 1.09; 95% CI: 0.86-1.37) did not show any statistically significant relationship. In terms of the kind of treatment setting at admission, there was no statistically significant relationship.

## Discussion

Our study shows that 12.67% of the study population in Georgia reported meth use, with the highest prevalence (8.05%) among individuals aged 25-49 years, which is similar to the trends in the literature [9]. Meth use prevalence is higher among certain racial groups, with Black or African American individuals (15.65%) and White individuals (10.76%) showing relatively higher rates. Gender does not seem to significantly affect meth use prevalence, as there is a relatively small difference between males (4.78%) and females (5.23%) in the reported use category. Notably, our study also indicates that the meth use pattern is more common in younger adults. This finding is similar to evidence in the literature that reported meth is most common among young adults aged 25-34 years, which is a cause for concern as this age group is more likely to experience long-term adverse effects [15].

Similarly, certain racial groups, such as Black or African American, have significantly higher odds of getting treatment for meth use compared to Asian or Pacific Islanders. Hence, highlights potential racial bias in meth use and access to treatment. Furthermore, lower education levels were associated with a higher likelihood of getting treatment for meth use, in contrast to individuals with higher education (four years of college or more). In addition, being employed full-time or part-time does not significantly affect the odds of getting treatment for meth use compared to being unemployed. However, not being in the labor force showed significantly lower odds of receiving treatment. Furthermore, being separated from marriage and receiving public financial assistance were associated with receiving treatment for meth use. Our study did not show any association regarding accessibility to treatment facilities. Another important finding from our study is the sizeable fraction of the population (80.37%) that lacks health insurance, thus affecting access to healthcare services, including addiction treatment.

Jones et al. (2022) stated that there are numerous effects of the rising prevalence of meth use, which could be short-term or long-term as chronic use can result in addiction, impair cognitive skills, and aggravate mental health conditions [9]. Other evidence in the literature has noted associated risky behaviors such as unsafe sexual practices and criminal activity, which leads to an increase in infectious diseases and pressure on law enforcement and healthcare systems [16]. Hence, preventive measures, treatment, and harm reduction methods are needed to curb against the elevated meth use in Georgia. Thus, justifying the importance of this study to gain a robust understanding of disparate bias in population attributes that will help to inform the public and policy decisions about the risks associated with meth use, and access to treatment.

Additionally, evidence in the literature has also shown that using meth has serious social and health repercussions, which can affect both the physical and mental health of the population [17]. The use of meth can result in elevated blood pressure, heat, decreased hunger, accelerated heart rate, arrhythmias, and stroke, which will affect the physical health of the individuals. Due to poor oral hygiene and the drug's acidic characteristics, prolonged use can also result in severe tooth issues. Meth use is also linked to risky behaviors that raise the chance of developing infectious diseases, such as unprotected sexual activity and sharing needles when taking drugs. To create effective preventive and treatment plans and address the broader societal effects of meth use, it is crucial to comprehend these implications, and the role of disparity. Similarly, meth use can have severe impacts on people's interpersonal connections, resulting in family dissolution, domestic violence, and child neglect [18]. Furthermore, meth use can lead to drug-related crimes like theft, burglary, and drug trafficking, which subsequently impact individuals, law enforcement, and the criminal justice system [19]. Additionally, meth has a severe negative impact on mental health in the form of anxiety, paranoia, hallucinations, and aggressive behavior, which further burdens the mental health system [20].

Limitations

The research faced several limitations that affected its findings. Some of the limitations included self-report bias. The data were collected via surveys and interviews and hence might have been subject to self-report bias, where participants may have underreported or over-reported their meth use due to social desirability or fear of judgment. The multiple regression utilized in the analysis was used to control for any bias that may have been identified in the study. Furthermore, while efforts were made to ensure a representative sample, specific population subgroups might have needed to be more represented or excluded from the study, limiting the generalizability of the findings to the entire population of Georgia State. Data analysis involves subjectivity, and different researchers may interpret the data differently. However, steps were taken to ensure rigor and inter-coder reliability, but some subjectivity may remain. Furthermore, the study's correlational nature somehow hindered establishing causal relationships between meth use and other factors. Other confounding variables may have been at play, influencing the observed associations. The study may not have captured all factors influencing the rise of meth use in Georgia State, as some relevant data were not available or accessible.

## Conclusions

Our study shows that there is a high prevalence of meth use in Georgia with existent sociodemographic disparities in accessing treatment. This study is of utmost importance, considering the alarming prevalence of this illicit drug and its associated health and social consequences in Georgia. Through a well-structured research methodology, the study shed light on the extent of meth use, existent sociodemographic differences, predicted vulnerable populations, and explored potential risk factors contributing to the high prevalence. This study also provided a comprehensive understanding of the trends and patterns of meth use treatment in Georgia. The findings will contribute to a deeper understanding of the factors affecting accessibility of treatment, thus guiding the development of targeted prevention, intervention, and other strategies to mitigate this inequity. Finally, the insights gained from this research will aid in mitigating the adverse health and social consequences of meth use.
